# Interferon-Stimulated Genes and Immune Metabolites as Broad-Spectrum Biomarkers for Viral Infections

**DOI:** 10.3390/v17010132

**Published:** 2025-01-18

**Authors:** Chien-Hsin Huang, Maudry Laurent-Rolle, Tyler L. Grove, Jack Chun-Chieh Hsu

**Affiliations:** 1 Center for Virus-Host-Innate-Immunity, Institute for Infectious and Inflammatory Diseases, New Jersey Medical School, Rutgers, The State University of New Jersey, Newark, NJ 07103, USA; ch1249@njms.rutgers.edu; 2 Section of Infectious Diseases, Department of Internal Medicine, Yale University School of Medicine, New Haven, CT 06520, USA; maudry.laurent-rolle@yale.edu; 3 Department of Microbial Pathogenesis, Yale University School of Medicine, New Haven, CT 06520, USA; 4 Department of Biochemistry, Albert Einstein College of Medicine, Bronx, NY 10461, USA; tyler.grove@einsteinmed.edu; 5 Department of Medicine, New Jersey Medical School, Rutgers, The State University of New Jersey, Newark, NJ 07103, USA

**Keywords:** Biomarker, type I Interferon, ISGs, ddhC, MxA, ISG15, IFI27, IFI44L, CXCL10

## Abstract

The type I interferon (IFN-I) response is a critical component of the immune defense against various viral pathogens, triggering the expression of hundreds of interferon-stimulated genes (ISGs). These ISGs encode proteins with diverse antiviral functions, targeting various stages of viral replication and restricting infection spread. Beyond their antiviral functions, ISGs and associated immune metabolites have emerged as promising broad-spectrum biomarkers that can differentiate viral infections from other conditions. This review provides an overview of the diagnostic potential of ISGs at transcript and protein levels, as well as their immune metabolites. We focus on their clinical applications and the sensitivity and specificity of these biomarkers through receiver operating characteristic (ROC) analysis. We highlight the need for further research to facilitate the effective translation of these biomarkers into clinical practice.

## 1. Introduction

Viral diseases continue to pose a major threat to public health, agriculture, livestock, and global economies. Recent outbreaks, including Zika, influenza, Ebola, and COVID-19, have highlighted the critical need for comprehensive strategies to manage viral diseases and mitigate their socioeconomic impact. Traditional approaches to managing viral diseases often focus on targeting virus-specific components. Diagnostic techniques such as PCR-based molecular tests and serological assays are designed to detect specific viral markers. These conventional approaches are highly effective for known pathogens but do not address newly emerging or rapidly mutating viruses, delaying timely response and exacerbating the healthcare burdens.

Broad-spectrum biomarkers offer a transformative solution by leveraging common host immune responses, rather than targeting virus-specific components. These biomarkers have the potential to differentiate viral infections from bacterial infections, even in cases involving novel pathogens, enabling faster medical responses and improved outbreak containment. This can facilitate more effective patient triage, better management of viral infections, and improved antibiotic stewardship. The clinical applications of broad-spectrum biomarkers extend to improving surveillance efforts for emerging and re-emerging viral threats, where rapid diagnostics are essential for outbreak control. Several host factors, including C-reactive protein (CRP), procalcitonin (PCT), mid-regional proadrenomedullin (MR-proADM), presepsin, ferritin, kynurenine, miRNAs, and host gene signatures, have demonstrated potential in differentiating viral infections from other conditions and predict disease outcomes [1,2,3,4,5,6,7,8,9,10]. CRP and PCT are commonly elevated in bacterial infections and are widely used as diagnostic biomarkers to distinguish bacterial from viral infections. However, both markers can also be elevated in severe respiratory viral infections, such as those caused by SARS-CoV-2 [11,12,13,14]. CRP, which is primarily used to differentiate bacterial from viral infections, demonstrates moderate diagnostic performance with receiver operating characteristic curve (AUC) values ranging from 0.6 to 0.85 across various studies, highlighting its limited accuracy as a standalone marker [15,16]. Nevertheless, its diagnostic utility can be significantly improved when combined with additional parameters. Additionally, emerging biomarkers like MR-proADM and presepsin have shown promise in sepsis diagnosis [17]. Both markers may also be elevated in severe viral infections and can serve as prognostic indicators in these cases [8,9,10]. Given the limited accuracy of traditional biomarkers, there is a critical need to develop novel strategies and identify new biomarkers to enhance the differentiation of viral infections from other pathologies.

To highlight their diagnostic potential, this review includes the AUC values for each marker, highlighting their sensitivity and specificity in identifying viral diseases. Sensitivity reflects the proportion of true positives among individuals with the target condition, including true positives and false negatives. Specificity indicates the proportion of true negatives among individuals without the condition, including true negatives and false positives. An AUC curve is generated by selecting various threshold values from continuous data. Changing these thresholds alters sensitivity and specificity, shaping the curve. Typically, as sensitivity increases, specificity decreases. An AUC value of 1 represents perfect discrimination between viral infections and other etiologies. An AUC between 0.7–0.8 is considered acceptable, with higher values indicating greater diagnostic potential. An AUC close to 0.5 suggests no discriminatory ability [18,19]. The selection of threshold values for biomarkers depends on their intended purpose, such as diagnosis, prognosis, or monitoring. Since sensitivity and specificity do not simultaneously increase or decrease, a compromise is necessary to determine the most appropriate threshold. For highly infectious and transmissible viruses, sensitivity is often prioritized over specificity to ensure effective disease control. In contrast, for low-prevalence diseases, specificity is emphasized to avoid unnecessary use of healthcare resources [20]. This review focuses on host-derived interferon-stimulated genes (ISGs), their proteins, and immune metabolites as a promising class of broad-spectrum biomarkers. Several ISGs, such as MxA, ISG15 and IFI27, exhibit unique expression patterns and robust diagnostic performance in identifying viral infections. A list of these potential biomarkers is summarized in Table 1.

## 2. Type I Interferon Response

**Figure 1:**
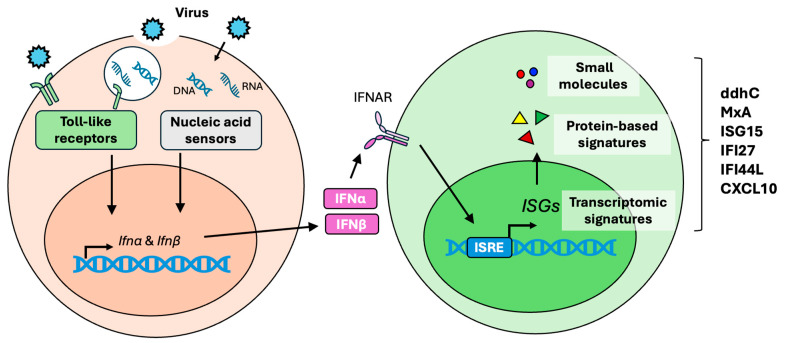
The IFN-I response and IFN-I-derived biomarkers. During viral infections, host cells recognize viral components through pattern recognition receptors, including toll-like receptors (TLRs) on the cell surface or in the endosomes. Additionally, viral DNA or RNA in the cytoplasm can be detected by nucleic acid sensors, such as RIG-I, MDA5, and cGAS. These sensors activate the induction and secretion of IFN-I (IFNα and IFNβ). The binding of IFNα and IFNβ to the type I interferon receptor (IFNAR) triggers the expression of interferon-stimulated genes (ISGs) driven by the interferon-stimulated response element (ISRE). Several ISGs, along with their protein products and small-molecule immune metabolites, have been identified as promising biomarkers for viral infections.

The type I interferon (IFN-I) response is an important antiviral defense mechanism triggered by viral infections (Figure 1). IFN-I, mainly IFNα and IFNβ, are the key mediators in coordinating antiviral responses. Most somatic cell types can produce IFN-I upon viral stimulation. In addition to IFN-I, type II IFN (IFN-II), represented solely by IFNγ, is essential in host defense. Unlike IFN-I, which is broadly produced by somatic cells, IFNγ is primarily secreted by immune cells such as T cells and natural killer (NK) cells. Through distinct signaling mechanisms, IFNγ is critical for viral clearance [55,56]. This review focuses exclusively on the IFN-I response.

During viral infection, the IFN-I response is activated when viral components, known as pathogen-associated molecular patterns (PAMPs), such as viral surface glycoproteins, DNA, and RNA, are recognized by host pattern recognition receptors (PRRs). For example, toll-like receptors (TLRs) localized in the endosomes, like TLR7 and TLR8, detect single-stranded RNA, TLR3 recognizes double-stranded RNA, and TLR9 recognizes CpG DNA. In the cytoplasm, several nucleic acid sensors are responsible for detecting viral DNA and RNA. Retinoic acid-inducible gene I (RIG-I) and melanoma differentiation-associated gene 5 (MDA5) recognize uncapped RNA and double-stranded RNA, while cyclic GMP-AMP synthase (cGAS) recognizes cytosolic DNA.

The activation of these PRRs triggers signaling pathways that involve key factors such as interferon regulatory factor 3 (IRF3) and TRAF family member-associated NF-κB activator (TANK)-binding kinase 1 (TBK1). Additionally, cGAS-mediated DNA sensing activates the stimulator of the interferon genes (STING) pathway [57,58]. These signaling cascades lead to the induction and secretion of IFNα and IFNβ. Once released, these IFNs engage in autocrine or paracrine signaling by binding to the IFN-I receptor (IFNAR) on the cell surface, activating the JAK-STAT signaling pathway. Specifically, IFN-I signaling through IFNAR triggers the phosphorylation of STAT1 and STAT2, which then associate with IRF9 to form the interferon-stimulated gene factor 3 (ISGF3) complex. This ISGF3 complex translocates to the nucleus, where it binds to interferon-stimulated response elements (ISREs) within DNA sequences. This binding drives the transcription of hundreds of interferon-stimulated genes (ISGs) [59], which encode antiviral proteins essential for establishing an antiviral state in host cells [60,61]. These ISGs are critical for orchestrating robust antiviral responses to contain and eliminate viral infections.

Given their unique expression patterns during viral infections, ISGs have been considered biomarkers at both protein and transcript levels. More recently, their associated immune metabolites have emerged as promising small-molecule biomarkers, further broadening their diagnostic potential. These developments position ISGs and their metabolites as critical tools for advancing the detection and management of viral infections.

## 3. ddhCTP Derivatives

**Figure 2:**
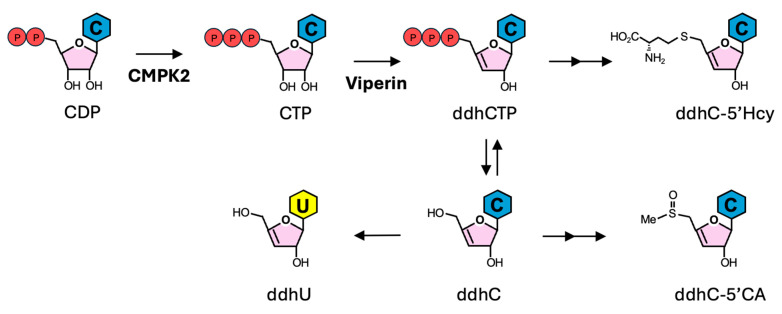
CMPK2, viperin, and the synthesis of ddhCTP-derived nucleoside analogs. The antiviral ISG protein UMP-CMP kinase 2 (CMPK2) catalyzes the phosphorylation of cytidine diphosphate (CDP) to produce cytidine triphosphate (CTP), which is then converted into 3′-deoxy-3′,4′-didehydro-CTP (ddhCTP) by the ISG protein viperin [65]. ddhCTP can be further converted into several ddhCTP-derived nucleoside analogs, including ddhC, ddh-uridine (ddhU), ddhC-5′-carboxylic acid (ddhC-5′CA), and ddhC-5′homocysteine (ddhC-5′Hcy). These four metabolites are the most highly induced nucleoside analogs in the urine and serum of patients with acute COVID-19 [22]. C, cytosine (in blue); U, uracil (in yellow).

3′-deoxy-3′,4′-didehydro-cytidine (ddhC), a nucleoside analog derived from ddhC triphosphate (ddhCTP), is an emerging biomarker with promising potential for differentiating viral infections from bacterial infections and non-infectious illness in humans. ddhCTP is synthesized by viperin, an antiviral protein viperin encoded by *RSAD2*, one of the most highly induced ISGs during viral infections [62]. Viperin plays a crucial role in the innate immune response to viral infections, restricting the replication of a broad spectrum of viruses, including human cytomegalovirus (HCMV), herpes virus 8 (HHV-8), Kaposi’s sarcoma-associated herpesvirus (KSHV), human immunodeficiency virus 1 (HIV-1), influenza A virus (IAV), hepatitis C virus (HCV), dengue virus (DENV), tick-borne encephalitis virus (TBEV), Zika virus (ZIKV), West Nile virus (WNV), and chikungunya virus (CHIKV), as previously reviewed [63]. As a member of the radical S-adenosyl methionine (SAM) superfamily of enzymes, viperin catalyzes the conversion of cytidine triphosphate (CTP) to ddhCTP (Figure 2) [64]. Notably, ddhCTP disrupts viral replication by two distinct mechanisms of action. First, ddhCTP targets viral RNA-dependent RNA polymerases, leading to the premature termination of viral RNA synthesis [65]. Second, ddhCTP activates a ribosome collision-dependent signaling cascade, leading to the inhibition of viral protein synthesis [64]. Additionally, another antiviral ISG, UMP-CMP kinase 2 (CMPK2), plays a critical role in ddhCTP synthesis by providing the substrate CTP for viperin [65]. CMPK2 is a nucleoside monophosphate kinase that primarily catalyzes the conversion of cytidine diphosphate (CDP) into cytidine triphosphate (CTP) (Figure 2) [65]. CMPK2 has been shown to protect host cells against virus infections through distinct mechanisms. CMPK2 inhibits ZIKV replication by translation inhibition, independent of its kinase activity [66]; in contrast, CMPK2 restricts coronavirus replication in a viperin-dependent manner [67].

Beyond its antiviral activity, the unique presence of ddhCTP during viral infection has significant potential in biomarker development. Recently, a group of nucleoside derivatives of ddhCTP have shown promise as biomarkers for viral infections (Figure 2). Through unbiased metabolomic analysis using liquid chromatography coupled with tandem mass spectrometry (LC-MS/MS) and nuclear magnetic resonance (NMR), Mehta et al. showed that one of these ddhCTP-derived nucleoside analogs, ddhC, was significantly upregulated in the serum samples from patients infected with a broad array of viruses, including SARS-CoV-2, IAV, influenza B virus (IBV), measles virus (MV), adenovirus, DENV, rotavirus, varicella-zoster virus (VZV) and respiratory syncytial virus (RSV) [21]. Notably, ddhC levels remain unchanged or are lower in patients with Gram-positive and Gram-negative bacterial infections or other non-infectious illnesses [21]. Although other studies have shown that viperin and CMPK2 are induced by lipopolysaccharides and bacterial infections [68,69], ddhC is found exclusively in viral-infected patients. This may be due to the involvement of additional host factors and/or virus-specific ISGs in ddhC synthesis. The serum level of ddhC correlates with the expression of *RSAD2* in blood. The performance of ddhC in differentiating viral-infected individuals from all other groups (bacterial-infected patients, non-infections unwell individuals, and healthy controls) achieved an AUC of 0.954, with a sensitivity of 0.881 and a specificity of 0.917. In addition, ddhC outperformed in differentiating viral infections from all other groups compared to CRP, white cell count, and lymphocyte count [21].

Recently, using NMR and LC-MS/MS analyses, several ddhCTP-derived nucleoside analogs have been detected to be up-regulated in serum and urine samples from COVID-19 patients [22]. These include deoxydidehydronucleosides (ddhN) derivatives, such as ddhC and ddh-uridine (ddhU) (Figure 2). These ddhNs can be detected in the urine four to five days after SARS-CoV-2 infection [22]. Notably, detecting ddhCTP-derived nucleoside analogs in urine samples provides a potential avenue for developing non-invasive biomarkers for viral infections.

## 4. MxA

Myxovirus resistance proteins, encoded by myxovirus resistance genes, are ISGs induced by type I and III IFNs [70,71]. These proteins play a critical role in antiviral immunity, particularly through the actions of MxA (also known as Mx1 in murine). MxA is a dynamin-like GTPase with broad-spectrum antiviral activity, which inhibits a wide variety of viruses, including IAV, MV, hepatitis B virus (HBV), and vesicular stomatitis Indiana virus (VSV) [72,73,74,75,76]. MxA exerts its antiviral effects by disrupting viral replication at multiple stages, including viral transcription, translation, assembly, and transport of viral components in host cells [70,77,78,79].

In addition to its broad-spectrum antiviral activity, MxA has emerged as a promising biomarker for viral infections. Although MxA is primarily an intracellular protein, it can be detected in the serum of children with respiratory viral infections with various viruses, including influenza virus, parainfluenza virus, adenovirus, rotavirus, rhinovirus, and RSV [24,25,26,30,31]. Higher MxA expression has been observed in children with viral infections compared to those with bacterial infections. The diagnostic performance of MxA showed an AUC of 0.89, with a sensitivity of 0.964 and a specificity of 0.667, in differentiating viral infections from bacterial infections [26]. Moreover, MxA also shows potential in differentiating viral infections from bacterial infections in adults. Analysis of MxA expression on monocytes in adults using flow cytometry revealed higher mean fluorescence intensity (MFI) in participants with viral infections compared to those with bacterial infections. To analyze MxA levels via flow cytometry, patient whole-blood samples were processed to isolate the cell pellet, which was then fixed and stained with anti-MxA antibodies. Flow cytometry was used to identify monocytes and measure their MFI of MxA. This yielded an AUC of 0.9, with a sensitivity of 0.923 and a specificity of 0.846 [29]. Additionally, MxA has demonstrated potential as a biomarker for COVID-19, effectively discriminating COVID-19 patients from those with bacterial infections and non-infected individuals in emergency department settings [80]. Notably, combining MxA and CRP levels has demonstrated improved diagnostic accuracy. With MxA predominantly elevated in viral infections and CRP levels higher in bacterial infections, the ratio of MxA to CRP has been proven to improve the accuracy of differentiating these infections. The combination of MxA and CRP significantly enhanced diagnostic performance, achieving an AUC of 0.92, with a sensitivity of 0.846 and a specificity of 1 [29].

MxA is predominantly expressed through IFN-dependent stimulation rather than direct viral stimulation [81]. As a result, it has been used to monitor IFN-I activity in conditions characterized by aberrant IFN-I responses, such as Sjögren’s syndrome, systemic lupus erythematosus, and systemic sclerosis [27,28]. Notably, some patients with *Streptococcus pneumoniae*, a Gram-positive bacterial infection, were found to have elevated MxA levels in the blood [26]. However, it remains unclear whether these individuals were co-infected with a virus. Given that MxA is also increased in certain non-infectious conditions, its diagnostic potential for viral infections requires further investigation.

## 5. ISG15

Interferon-stimulated gene 15 (ISG15) is a ubiquitin-like protein that plays a critical role in the innate antiviral response. ISG15 can inhibit the replication of a wide range of viruses, including IAV, IBV, herpes simplex virus type 1, Sindbis virus, Ebola virus, CHIKV, murine norovirus, DENV, and WNV [82,83]. ISG15 can be conjugated to both host and viral proteins through a process known as ISGylation, which has been shown to restrict several viral infections. In addition to its ISGylation-dependent effects, ISG15 can also inhibit viral replication through ISGylation-independent mechanisms, highlighting its multifaceted role in antiviral immunity, as previously reviewed [82,84].

ISG15 is one of the most highly induced ISGs during the IFN-I response. *ISG15* has been implicated in various biomarker panels for differentiating viral and bacterial infections. A four-gene blood signature that includes *ISG15*, *IL16*, 2′,5′-*O*ligoadenylate *S*ynthetase *L*ike (*OASL*), and *Ad*hesion *G* protein-coupled *R*eceptor *E5* (*ADGRE5*) yielded an AUC of over 0.90 in distinguishing various viral infections from non-viral conditions [32]. Additionally, a 10-gene panel, also featuring *ISG15*, can differentiate viral from bacterial infections with an AUC of 0.97, with a sensitivity of 0.93 and a specificity of 0.97 [33]. Notably, in COVID-19 patients, protein ISGylation is elevated in the peripheral blood mononuclear cells (PBMCs) isolated from symptomatic patients, but it remains unchanged in both asymptomatic and uninfected individuals [85].

Beyond its critical role in innate antiviral responses, ISG15 is also induced and involved in cancer [34], emphasizing its significance in immune regulation. The study of ISG15 not only enhances our understanding of antiviral mechanisms but also holds broader implications for the development of diagnostic tools for a range of diseases. Further investigation is required to fully understand the specificity and clinical applicability of ISG15, which will be crucial for its effective integration into clinical practice.

## 6. IFI27

Interferon alpha-inducible protein 27 (IFI27), also known as ISG12a, has antiviral activity against HCV [86]. *IFI27* is upregulated in blood samples during viral infections and has emerged as a robust single-gene biomarker. Detecting *IFI27* mRNA in peripheral blood can differentiate influenza from bacterial infection with an AUC of 0.91, with a sensitivity of 0.80 and a specificity of 0.90 [35]. Moreover, using an in vitro peripheral blood culture system, plasmacytoid dendritic cells (pDCs) were identified as the primary sources of *IFI27* in peripheral blood, primarily driven by TLR7 activation [35]. Beyond influenza, *IFI27* has been used to predict outcomes in patients infected with various viruses, including RSV, SARS-CoV-2, and enterovirus 71 [36,37,38,87]. Additionally, *IFI27* has been integrated into various biomarker panels as a multi-gene biomarker. A 10-gene panel, including *IFI27*, distinguishes viral from bacterial infections with an AUC of 0.97, with a sensitivity of 0.93 and a specificity of 0.97 [33]. A seven-gene panel, also featuring *IFI27*, demonstrated similar accuracy in differentiating between viral and bacterial infections, with an AUC of 0.91 [88,89]. Additionally, since intracellular bacterial infections can trigger IFN responses similar to viral infections, an eight-gene signature was developed to distinguish intracellular and extracellular bacterial infections from viral infections. This panel, including *IFI27*, showed an AUC of over 0.90 in various comparisons, including extracellular versus viral infections, intracellular bacterial versus viral infections, and all bacterial versus viral infections [90].

## 7. IFI44L

Interferon-induced protein 44-like (IFI44L) is an antiviral ISG with mechanisms of action dependent on the specific context of viral infections. IFI44L effectively inhibits RSV replication in vitro [91], whereas it demonstrates moderate antiviral activity against HCV in an in vitro overexpression screen [92]. However, IFI44L has a negative role in the antiviral response against HBV and IAV [93,94]. *IFI44L* is upregulated in the blood of viral-infected patients and has been proposed as a promising diagnostic biomarker for viral infections [39,40,95]. *IFI44L* was elevated in viral-infected children compared to healthy children [40]. The proposed two-transcript panel, combining *IFI44L* with *FAM*ily with sequence similarity 89 *M*ember *A* (*FAM89A*), which is increased in bacterial infections, demonstrated an AUC of 0.974, with a sensitivity of 1.0 and a specificity of 0.964 in distinguishing viral from bacterial infections [40]. Additionally, *IFI44L* has shown potential as a biomarker in adults for differentiating viral from bacterial infections. A two-transcript signature, comprising *IFI44L* and *P*eptidase *I*nhibitor 3 (*PI3*), achieved an AUC over 0.90 [39].

## 8. CXCL10

C-X-C motif chemokine ligand 10 (CXCL10), also known as IFN gamma-induced protein-10 (IP-10), is an ISG induced during inflammatory conditions by type I and II IFNs. As a pro-inflammatory chemokine, CXCL10 plays an important role in the antiviral immune response, recruiting immune cells to sites of infection or inflammation. CXCL10 primarily binds to the CXCR3 receptor, which is highly expressed in T cells. This CXCL10/CXCR3 signaling axis is implicated in various DNA and RNA virus infections [96]. In mouse models infected with mouse hepatitis virus (MHV) or WNV, CXCL10 stimulation in T cells is essential for effective viral clearance [97,98]. Additionally, elevated levels of *CXCL10* have been detected in human samples from infections with various viruses, including ZIKV, DENV, MV, HIV-1, and several respiratory viruses [41,42,43,44,50,99,100]. As a result, *CXCL10* has been identified as a promising biomarker for viral infections. For instance, the RNA levels of *CXCL10* were significantly elevated in nasopharyngeal samples from individuals infected with respiratory viruses. When combined into a three-gene panel with *IFIT2* and *OASL*, the ROC analysis yielded an AUC of 0.97 [42]. Additionally, the protein levels of CXCL10 alone achieved an AUC of 0.85 in nasopharyngeal samples [42].

In COVID-19 patients, elevated CXCL10 levels are linked to disease severity and serve as a robust predictive biomarker for disease progression and mortality, while decreasing levels indicate clinical improvement. This highlights its effectiveness in assessing patient outcomes, as previously reviewed [101]. For example, plasma CXCL10 was identified as a key predictor for ICU transfer and mortality in COVID-19 patients, with an AUC of 0.8374 when combined with the neutrophil-to-lymphocyte ratio (NLR) and time from symptom onset and with an AUC of 0.7334 when used alone to predict death [46]. Furthermore, CXCL10, along with *T*umor necrosis factor-*R*elated *A*poptosis-*I*nducing *L*igand (TRAIL) and CRP levels in the blood, forms a powerful host-derived signature for differentiating viral from bacterial infections, demonstrating a high AUC in the diagnosis and prognosis of these infections [102,103,104].

Increased levels of CXCL10 have been observed in the plasma and serum of individuals infected with various bacteria and parasites [47,48,49,51,52,53,54]. Additionally, serum CXCL10 is also evaluated in both infectious and non-infectious conditions, including sepsis, autoimmune diseases, and cancers, as previously reviewed [101]. Therefore, further investigation is required to understand the specificity and clinical applicability of CXCL10 fully.

## 9. Practical Considerations for Clinical Application

The high AUC values of these biomarkers highlight their potential to distinguish viral infections from other conditions in specific contexts. However, there are still some limitations to their use in clinical practice. First, some markers exhibit non-specific expression and may also be induced in response to bacterial pathogens or non-infectious causes. This highlights the need for robust diagnostic strategies to enhance specificity. Potential solutions include leveraging machine learning algorithms to optimize cutoff values, incorporating clinical metadata, or combining biomarkers into multiplex panels for greater accuracy. While these markers may increase in multiple conditions, the level of expression is often distinct. For example, ddhC shows significant elevation in viral infections but only minor increases in bacterial infections caused by pathogens like *Streptococcus pneumoniae* or *Klebsiella pneumoniae*. These slight increases are much less pronounced compared to those seen in viral infections such as SARS-CoV-2, emphasizing the importance of determining reliable cutoff values for diagnostic use. Second, the performance of biomarkers may vary across populations and disease contexts. Rigorous validation studies are essential to address this variability. Such studies should encompass diverse demographics, comorbidities, and geographic regions to ensure their clinical applicability and consistency. Furthermore, incorporating real-world data into validation studies can help refine diagnostic protocols and account for variability in disease presentations. Third, the expression levels of some biomarkers may be too low to detect in patients with mild or early-stage disease, potentially limiting their application in asymptomatic or mild symptomatic cases. To address this, combining these biomarkers with highly sensitive detection technologies or integrating them into panels with complementary markers may improve diagnostic capabilities. Advanced analytical methods, such as integrating multi-omics approaches, could further enhance sensitivity and specificity. Lastly, ELISA, RT-qPCR, RNA-Seq, and LC-MS/MS are widely used biomarker detection methods, each with unique strengths and limitations affecting diagnostic performance. ELISA is simple, cost-effective, and suitable for routine use but may struggle with low-abundance biomarkers due to its dependency on antibody quality. RT-qPCR, a gold standard for nucleic acid detection, provides high sensitivity and specificity but requires high-quality RNA, advanced reagents, and equipment. Methods like RNA-Seq and LC-MS/MS offer unparalleled specificity and the ability to analyze diverse targets. However, they are time-intensive, costly, and require specialized expertise, making them less practical for rapid diagnostics. Although ELISA and RT-qPCR are more accessible for routine and resource-limited settings, RNA-seq and LC-MS/MS remain invaluable for research and high-precision diagnostics. To address variability and enhance clinical integration, standardized protocols are essential to ensure reproducibility, particularly when transitioning from research to practice. Despite these limitations, the continued development of these biomarkers through extensive validation and innovative diagnostic methodologies holds promise for their effective clinical integration.

## 10. Conclusions

The IFN-I response is a cornerstone of the immune defense against viral infections, inducing the expression of ISGs to restrict viral replication and spread. As the global burden of viral diseases continues to rise, particularly with new and re-emerging pathogens, the need for rapid, accurate diagnostic tools is more critical than ever. This review highlights the potential of ISGs, their encoded proteins, and associated immune metabolites as broad-spectrum biomarkers that could improve the diagnosis and management of viral infections.

We focus on various biomarkers, including ddhCTP derivatives, MxA, ISG15, IFI27, IFI44L, and CXCL10, emphasizing their diagnostic applications in distinguishing viral infections from bacterial infections and other non-infectious conditions, as well as their predictive roles in monitoring disease progression in viral infections. The unique expression patterns and functional roles of these ISGs make them valuable candidates for developing broad-spectrum biomarkers. The emerging evidence on immune metabolites, such as ddhCTP derivatives, further expands the diagnostic landscape, offering non-invasive options that could enhance early detection and improve patient outcomes.

While these biomarkers demonstrate considerable promise, further research is essential to refine their specificity, sensitivity, and clinical applicability. Advanced approaches, such as the integration of multiplex biomarker panels, machine learning, and validation studies in diverse populations, will be key to overcoming existing limitations. These efforts will ensure the effective incorporation of these biomarkers into routine clinical diagnostics. Integrating these biomarkers with conventional diagnostic methods could provide more robust, adaptable, and scalable diagnostic tools. Such advancements will enhance our preparedness for future viral threats and enhance our ability to respond to ongoing global health challenges.

## Figures and Tables

**Table 1:** Viral disease biomarkers derived from the type I interferon response.

Biomarker	Sample Source	Substance Type	Detection Approach	Pathogen and Etiology	Refs.
ddhC	Serum Urine	Nucleoside derivative	NMR LC-MS/MS	SARS-CoV-2 Influenza viruses Adenovirus Dengue virus Rotavirus Respiratory syncytial virus Varicella-zoster virus Measles virus	[21,22]
MxA	Blood Monocytes Lymphocytes	Protein	ELISA Immunochemiluminescent assay Flow cytometry	Rotavirus Respiratory syncytial virus Influenza virus SARS-CoV-2 Rhinovirus Human bocavirus Human metapneumovirus Parainfluenza virus Rhinovirus Adenovirus Enterovirus Herpes simplex virus Epstein–Barr virus Bacteria * Autoimmune diseases *	[23,24,25,26,27,28,29,30,31]
ISG15	Whole blood PBMCs	RNA Protein(ISGylation)	RNA-Seq	Rhinovirus Adenovirus Respiratory syncytial virus Influenza virus SARS-CoV-2 Enterovirus Human metapneumovirus HIV-1 Herpes virus Varicella-zoster virus Epstein–Barr virus Cytomegalovirus Dengue virus Cancer *	[32,33,34]
IFI27	Blood PBMCs	RNA	RT-qPCR	Influenza virus Enterovirus 71 Respiratory syncytial virus Human metapneumovirus Herpes virus Cytomegalovirus Adenovirus Rhinovirus SARS-CoV-2 Dengue virus	[33,35,36,37,38]
IFI44L	Blood	RNA	Reverse Transcriptase Loop-Mediated Isothermal Amplification (RT-LAMP) RT-PCR	Adenovirus Influenza virus Respiratory syncytial virus Rotavirus Enterovirus Epstein–Barr virus Epidemic hemorrhagic fever virus Severe fever with thrombocytopenia syndrome virus	[39,40]
CXCL10	Nasopharyngeal swab Plasma Serum Cerebrospinal fluid	RNA Protein	RT-qPCR Microbeads multiplex immunoassay ELISA Magnetic Luminex Assay Cytometric bead array	Adenovirus Human metapneumovirus Influenza virus Parainfluenza virus Respiratory syncytial virus Rhinovirus SARS-CoV-2 Zika virus Dengue virus Measles virus HIV-1 Bacteria * Parasites * Autoimmune diseases * Cancer *	[41,42,43,44,45,46,47,48,49,50,51,52,53,54]

* Biomarkers are also elevated in different etiologies.
